# Diversity and networking of uni-cyanobacterial cultures and associated heterotrophic bacteria from the benthic microbial mat of a desert hydrothermal spring

**DOI:** 10.1093/femsec/fiae148

**Published:** 2024-11-18

**Authors:** Khaoula Lassoued, Mouna Mahjoubi, Elias Asimakis, Naima Bel Mokhtar, Panagiota Stathopoulou, Refka Ben Hamouda, Olfa Bousselmi, Ramona Marasco, Ahmed Slaheddine Masmoudi, Daniele Daffonchio, George Tsiamis, Ameur Cherif

**Affiliations:** BVBGR-LR11ES31, ISBST, Biotechpole Sidi Thabet, Univ. Manouba, Ariana 2020, Tunisia; National Institute of Agronomy of Tunisia, University of Carthage, Tunis 1082, Tunisia; BVBGR-LR11ES31, ISBST, Biotechpole Sidi Thabet, Univ. Manouba, Ariana 2020, Tunisia; Laboratory of Systems Microbiology and Applied Genomics, Department of Environmental Engineering, University of Patras, Agrinio 30100, Greece; Laboratory of Systems Microbiology and Applied Genomics, Department of Environmental Engineering, University of Patras, Agrinio 30100, Greece; Laboratory of Systems Microbiology and Applied Genomics, Department of Environmental Engineering, University of Patras, Agrinio 30100, Greece; BVBGR-LR11ES31, ISBST, Biotechpole Sidi Thabet, Univ. Manouba, Ariana 2020, Tunisia; BVBGR-LR11ES31, ISBST, Biotechpole Sidi Thabet, Univ. Manouba, Ariana 2020, Tunisia; Biological and Environmental Sciences and Engineering Division (BESE), King Abdullah University of Science and Technology (KAUST), Thuwal 23955, Saudi Arabia; BVBGR-LR11ES31, ISBST, Biotechpole Sidi Thabet, Univ. Manouba, Ariana 2020, Tunisia; Biological and Environmental Sciences and Engineering Division (BESE), King Abdullah University of Science and Technology (KAUST), Thuwal 23955, Saudi Arabia; Laboratory of Systems Microbiology and Applied Genomics, Department of Environmental Engineering, University of Patras, Agrinio 30100, Greece; BVBGR-LR11ES31, ISBST, Biotechpole Sidi Thabet, Univ. Manouba, Ariana 2020, Tunisia

**Keywords:** thermal spring, cyanobacteria, heterotrophic bacteria, desert oasis, phycosphere, Ksar Ghilane

## Abstract

Thermal springs harbour microorganisms, often dominated by cyanobacteria, which form biofilms and microbial mats. These phototrophic organisms release organic exudates into their immediate surroundings, attracting heterotrophic bacteria that contribute to the diversity and functioning of these ecosystems. In this study, the microbial mats from a hydrothermal pool in the Ksar Ghilane oasis in the Grand Erg Oriental of the Desert Tunisia were collected to obtain cyanobacterial cultures formed by single cyanobacterial species. High-throughput analysis showed that while the microbial mat hosted diverse cyanobacteria, laboratory cultures selectively enriched cyanobacteria from the *Leptolyngbya, Nodosilinea*, and *Arthronema*. Per each of these genera, multiple non-axenic uni-cyanobacterial cultures were established, totalling 41 cultures. Cyanobacteria taxa mediated the assembly of distinct heterotrophic bacterial communities, with members of the Proteobacteria and Actinobacteria phyla dominating. The bacterial communities of uni-cyanobacterial cultures were densely interconnected, with heterotrophic bacteria preferentially co-occurring with each other. Our study highlighted the complex structures of non-axenic uni-cyanobacterial cultures, where taxonomically distinct cyanobacteria consistently associate with specific groups of heterotrophic bacteria. The observed associations were likely driven by common selection pressures in the laboratory, such as cultivation conditions and specific hosts, and may not necessarily reflect the microbial dynamic occurring in the spring microbial mats.

## Teaser

Investigation of the microbial diversity of cyanobacteria and heterotrophic bacteria associated with uni-cyanobacterial cultures obtained in laboratory from the microbial mats of a hydrothermal pool in the Sahara desert.

## Introduction

Thermal springs result from the geothermal heat generated by magma chambers beneath the Earth’s surface (McCall 2005), which elevates the temperature of groundwater well above the local ambient temperature (Najar et al. 2022). The unique conditions created by geothermal ecosystems, characterized by high (>50°C) or low-medium (40°C–50°C) temperatures and mineral-rich water, make these areas ideal for colonization by thermally adapted extremophile microorganisms, like thermophilic, hyperthermophilic, and thermoresistant bacteria (Shu and Huang 2022). Studies report that various factors, including the physical and chemical characteristics of the geothermal water, its origin, the nature of the geothermal gradient, and the internal pressure, collectively influence the versatility and heterogeneity of the microbial communities (Rampelotto 2013, Merino et al. 2019). Despite such variation, cyanobacteria are consistently found and tend to be the predominant components of the microbial communities in many thermal springs (Whitton and Potts 2000, Singh et al. 2018), forming, in many cases, unique biofilm structures. These structures are composed of gelatinous pellicles that can float at the surface or develop pelagically in sulfur-bearing spring water (Gomes et al. 2013, Biondi et al. 2023). Cyanobacteria, as primary producers, are also primarily responsible for forming microbial mats in these habitats (Mehdizadeh Allaf and Peerhossaini 2022). In such mats, the micro-environment surrounding the phytoplankton and phytobenthos cells (including those of cyanobacteria), referred to as the phycosphere (or specifically cyanosphere), serves as a dynamic interface where microorganisms, including heterotrophic bacteria, can engage in interaction with cyanobacteria and algae (Seymour et al. 2017). Various types of interactions, including symbiosis (Zehr 2015), mutualism (Buchan et al. 2014), parasitism, or competition (Amin et al. 2015, Durham et al. 2015), have been reported. These interactions can shape the structure and function of the ecosystems, impacting nutrient cycling, carbon fixation, and oxygen dynamics (Bagatini et al. 2014, Akins et al. 2018, Jankowiak and Gobler 2020, Zhao et al. 2023).

The complexity of the interactions occurring in microbial mat communities has led researchers to use simplified cyanobacterial consortia to better understand these dynamics. These communities, derived from natural environments, are obtained by the selective enrichment and isolation of single cyanobacterial species and their associated bacteria. This approach allows scientists to create simplified ecosystems that mimic and replicate certain aspects of the natural environment while offering a higher control in a laboratory setting (Paerl and Pinckney 1996, Paerl et al. 2000, Ataeian et al. 2022). For instance, cyanobacterial cultures with coexisting bacterial partners, constituting the so-called cyanosphere, exhibit greater stability, robustness, and longer lifespans compared to axenic cultures (Rippka et al. 1981, Morris et al. 2008, Katoh et al. 2012, Cole et al. 2014, Jackrel et al. 2021). This co-culturing strategy not only enables the investigation of the cyanobacteria-heterotrophic bacteria interactions but also gives the opportunity to address specific ecological questions and unravel mechanisms of early successional formation in complex aquatic systems, such as how both microbial components, i.e. cyanobacteria and heterotrophic bacteria, drive and influence the assembly and structure of cyanobacteria mat community.

In the relentless pursuit of understanding the role of thermal-adapted cyanobacteria in hot springs and their interaction with surrounding microorganisms, scientists have delved into some of the world’s most iconic geothermal landscapes. The microbial ecology of Yellowstone National Park in the USA has stood at the forefront of this exploration (Ward et al. 1998, Wood et al. 2024) setting the stage for a global journey into the depths of hot springs across continents, spanning from the North Chilean Patagonia (Mackenzie et al. 2013) to Yunnan Province in China (Keshari et al. 2022), Sembawang in Singapore (George et al. 2023), Malaysia (Chan et al. 2015), India (Subudhi et al. 2018), Japan (Nakagawa and Fukui 2002), Russia (Rozanov et al. 2017), and Greece (Kanellopoulos et al. 2022), as well as from the arid landscapes of North African Algerian (Amarouche Yala et al. 2014) and Tunisia (Sayeh et al. 2010).

However, the thermophilic cyanobacteria of geothermal springs in desert ecosystems have been poorly studied, even if understanding their microbial diversity and dynamics can not only enhance our knowledge of extremophilic life adaptability but also reveal the ecological equilibria, as well as the resilience of these unique ecosystems. Yet, by adapting to harsh environments, these bacteria generate distinct genetic features and metabolic pathways, which may be useful for a variety of commercial and biotechnological applications such as enzymes, proteins, or other biologically active compounds (Abed et al. 2009, Singh et al. 2018, Debnath et al. 2019). Here, we characterize the diversity of cyanobacteria in the hot spring of the Ksar Ghilane oasis located in the Grand Erg Oriental desert in the south of Tunisia and reveal the interactions between these photosynthetic bacteria and the associated heterotrophic bacteria. We hypothesized that cyanobacteria are important diversity components of the phycosphere of the hot spring, shaping the assembly and composition of their associated heterotrophic bacterial community. To do this, uni-cyanobacterial cultures (UCCs), i.e. complex communities in which a single cyanobacterium takes on the role of the primary autotrophic organism, were obtained from environmental samples. The UCCs were characterized by applying amplicon sequencing of the 16S rRNA gene and correlation-based network analysis to the overall cyanosphere, as well as by isolating the heterotrophic bacterial component.

## Materials and methods

### Sampling

**Figure 1.**
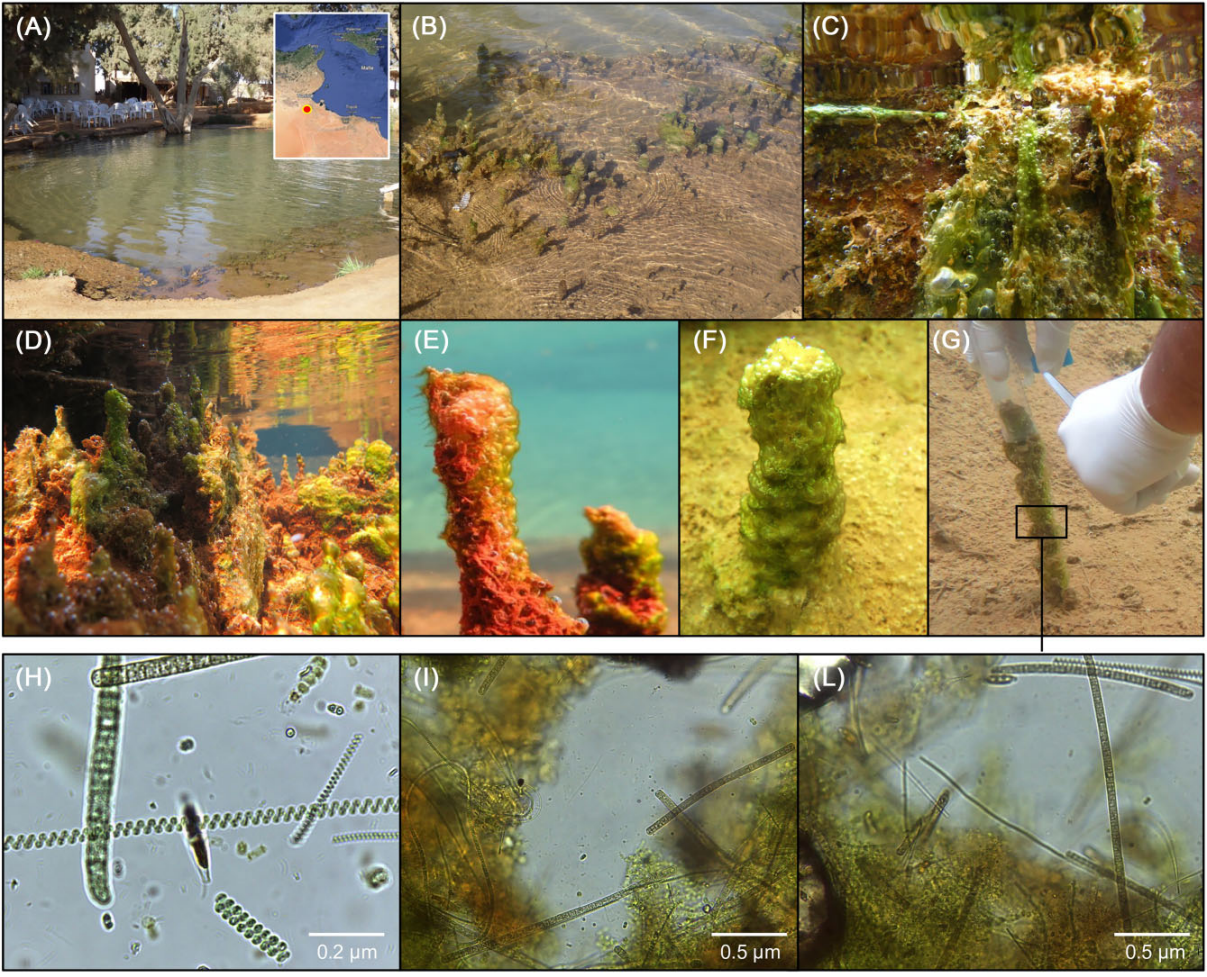
(A) Hydrothermal pool in the oasis of Ksar Ghilane, located in the Kebili region of Tunisia, at the border with the Sahara Desert (see map). (B–F) Representative pictures of the biofilm formation in the pool, including benthic and floating microbial mat structures of different colours, from green to red and light brown. (G) Sampling of the benthic microbial mat. (H–L) Microscopic observations of the collected microbial mats; additional images are reported in Supplementary Fig. S3.

The selected site is a hydrothermal pool (Fig. 1A) located in the Ksar Ghilane oasis in the southern region of Tunisia (Kebili, N 32°59.186′, E 008°38.241′). It is formed and fed by a source of hot spring water (Najjari et al. 2023), and it is characterized by the presence of several algal and cyanobacterial microbial mats (Fig. 1A–F). During an expedition under the Biodesert EU project in November 2016, water samples and three morphologically different microbial mats were collected aseptically into sterile tubes and polypropylene bags (Fig. 1G) and stored immediately at 4°C until reaching the laboratory. Water temperature, salinity, conductivity, and pH were determined using a multi-parameter probe. Physicochemical parameters of the water source were carried out using Merck Millipore^®^ test kit and WTW pHotoFlex^®^ spectrophotometer (Table 1).

**Table 1.** Physicochemical parameters of the water in the Ksar Ghilane pool.

Parameters	This study	Essamin and Kamel	Matsumoto et al.
Temperature (°C)	34	33.5	34
pH	7	7.6	7.1
Conductivity (ms cm^−1^)	4.52	NA	4.58
Hardness	NA	2010	2028
Sodium (mg l^−1^)	1138	807	740
Potassium (mg l^−1^)	75.6	31.7	53
Magnesium (mg l^−1^)	NA	222	224
Calcium (mg l^−1^)	NA	434	443
Sulfate (mg l^−1^)	292	1400	1410
Phosphate (mg l^−1^)	1.2	NA	NA
Salinity (ppt)	2.71	NA	NA
Nitrate (mg l^−1^)	<0.05	NA	NA
Nitrite (mg l^−1^)	<0.02	NA	NA
Aluminium (mg l^−1^)	70	NA	NA
TDS	NA	4365	4306
Turbidity (NTU)	<0.01	NA	NA

Values reported are obtained from measurements conducted in (i) this study (see the ‘Materials and Methods’ section), (ii) Essamin and Kamel (2018), and (iii) Matsumoto et al. (2020); NA, non-available.

### Cultivation and isolation of cyanobacteria from spring microbial mats

Cyanobacterial microbial mats were rinsed with sterile physiological saline solution (0.9% w/v NaCl) several times to eliminate the free-living bacteria and were then examined under a Leica ICC50 HD optical microscope to assess initial diversity. Starting from different portions of the collected microbial mats, a first enrichment was performed in liquid BG11 medium (Rippka et al. 1981, Rippka 1988, Waterbury 2006) supplemented with vitamin B_12_ (0.02 mg l^−1^). Cultures were incubated at 30°C under artificial optical radiation of 40 µmol photons m^−2^ s^−1^ emitted by LED tubes with 16:8 h light/dark photoperiods for 30–40 days. When growth was visible, subcultures were transferred in fresh liquid medium and agar plates (1% w/v agar). Serial dilutions and streak plate methods were used for single-cell isolation of cyanobacteria. Under a binocular microscope, individual cyanobacterial colonies and filaments were differentiated and transferred to a fresh medium. UCCs were then conserved in glycerol stock (25% v/v) and stored at −20°C for DNA extraction.

### DNA extraction

Total DNA was isolated from the environmental microbial mat samples and UCCs using the NucleoSpin™ Soil kit (Macherey-Nagel, Düren, Germany) according to the manufacturer’s instructions. Specifically, 0.5 g of microbial mat samples or 500 µl of UCCs were collected in 2 ml Eppendorf tubes. Mechanical cell lysis using glass beads (4 mm) was first applied to enhance the DNA extraction yield. DNA integrity, quantity, and purity of each sample were checked using agarose gel electrophoresis [0.8% (w/v) agarose in 1X TAE buffer (40 mM Tris-acetate, 1 mM EDTA)] and a Q5000 micro-volume UV–vis spectrophotometer (Quawell Technology, San Joe, CA, USA). DNA samples were stored at −20°C until further use.

### Library preparation and Illumina MiSeq sequencing

The total DNA extracted from the microbial mats and UCC samples was not sufficient to generate adequate amplificon product in a single-step Polymerase Chain Reaction (PCR) for downstream library preparation for Illumina MiSeq sequencing. To overcome this limitation, a nested PCR strategy was employed. PCRs were performed using the KAPA Taq polymerase kit (KAPA Biosystems, Wilmington, MA, USA). An initial round of PCR was performed using the primers 27F (Edwards et al. 1989) and 1492R (Weisburg et al. 1991) under the following thermal cycling conditions: an initial denaturation step at 95°C for 3 min, followed by 20 cycles of denaturation at 95°C for 30 s, annealing at 55°C for 30 s, and extension at 72°C for 1 min, with a final extension step at 72°C for 5 min. Each 25 µl reaction contained KAPA buffer at a final concentration of 1X, 0.2 mM of dNTPs, 0.2 µM of each primer, 0.5 U of KAPA Taq DNA polymerase, 5 µl of extracted DNA as template, and sterile deionized water. The product from this first PCR was then used as a template for the second round of PCR using universal fusion primers S-D-Bact-0341-b-s-17 and S-D-Bact-0785-a-A-2 (Herlemann et al. 2011, Klindworth et al. 2013) and the same reaction conditions and amplification protocol described above. The amplified fragments (450 bp) were finally visualized using the Bio-Rad Gel Doc XR+ system. Positive PCR products were purified using an equal volume of PEG solution [20% PEG 8000 (m/v), 2.5 M NaCl]. After incubation at 37°C for 15 min, centrifugation was performed at 14 000 × *g* for 20 min. The pellet was washed twice with 125 µl of 80% and 70% ethanol, respectively. Centrifugation at 4°C for 10 min was applied each time. The pellet was then dried at 37°C and suspended in 15 µl of sterile deionized water. The concentration was measured using a Q5000 micro-volume UV–vis spectrophotometer. The purified PCR products were diluted up to 10 ng/µl and used as templates for a next step PCR to include the indexes and the Illumina adaptors. The combinatorial use of index primers generated unique samples that were pooled and sequenced on one Illumina MiSeq run. PCR reaction was carried out in a final volume of 50 µl containing 1× of KAPA Taq Buffer, 0.3 mM dNTPs, 0.1 mM of the forward and the reverse indexing primers, 0.5 U of KAPA Taq DNA polymerase, 2 µl from the diluted PCR product, and sterile deionized water. The amplification protocol included 3 min incubation at 95°C followed by 8 cycles of 95°C for 30 s, 55°C for 30 s, and 72°C for 30 s, and a final elongation step at 72°C for 5 min. Amplicons were then purified using NucleoMag^®^ NGS Clean-up and Size Selection kit (Macherey-Nagel, Düren, Germany) according to the manufacturer’s recommendations and quantified using a Q5000 micro-volume UV–vis spectrophotometer. Purified PCR products were then combined in equimolar ratios (8 nM). High-throughput sequencing was performed by Macrogen Inc. (Seoul, Korea) on the Illumina MiSeq platform using a paired-end (2 × 300 bp) kit.

### Sequence data processing, bacterial diversity, and statistical analysis

Files containing raw sequencing reads were de-multiplexed and converted into FASTQ format. Adapters and indexes were trimmed using Trimmomatic v0.39 with a threshold quality score of Q15 (Bolger et al. 2014). Clustering, alignment, and phylogenetic analysis of 16S rRNA gene fragments were performed using USEARCH v.11 (Edgar 2010) and Qiime2 distribution 2019.1 (Bolyen et al. 2019). Forward and reverse reads of each sample were assembled into paired-end reads, trimmed by length, and combined in a single FASTQ file using the ‘*-fastq_mergepairs*’ command with ‘*-fastq_maxdiffs*’, ‘*-fastq_pctid*’, *‘-fastq_minmergelen*’, and ‘*-fastq_maxmergelen*’ options set at default values. Assembled reads were quality-filtered using ‘*-fastq_filter*’ command with the ‘*-fastq_maxee*’ option set at 1.0. Unique read sequences were obtained by removing redundant sequences with the ‘*-fastx_uniques*’ command. Subsequently, reads were clustered into operational taxonomic units (OTUs) at a 97% similarity cut-off by running the UPARSE algorithm (Edgar 2013) with the ‘*-cluster_otus*’ command. Cross-talk errors were filtered using the ‘*-uncross*’ command of the UNCROSS2 algorithm (Edgar 2018). Extremely rare OTUs that account for <0.001% of the total reads across all samples were excluded from the analysis using the ‘*-otutab_trim*’ command. Taxonomy was assigned using the algorithm ‘*-classify-sklearn*’ against the SILVA database 138 (Quast et al. 2012, Bokulich et al. 2018) trained on the V3–V4 region for the 16S rRNA gene. The EzTaxon BioCloud database (https://www.ezbiocloud.net) was further used to confirm the classification of cyanobacterial OTUs (Supplementary Data S1). Alpha diversity indices, along with metrics representing population structure (such as observed OTUs and good coverage), were calculated in MetaXplore (Bel Mokhtar et al. 2024), setting the rarefaction threshold at 50 000. Pairwise ANOVA was used to identify significant differences in alpha diversity indices between UCC groups. Rarefaction curves are reported in Supplementary Fig. S1. Canonical analysis of principal coordinates (CAP) (Anderson and Willis 2003) based on the Bray–Curtis similarity matrix was performed to establish the clusterization of bacterial communities based on the UCC groups established. Distance-based tests for homogeneity of multivariate dispersions (PERMDISP) and permutational multivariate analysis of variance (PERMANOVA) were further assessed with Primer 6 (Anderson 2001) to identify the significant differences among the groups. Upset plots were constructed using the UpSet package (Conway et al. 2017).

### Co-occurrence network of bacterial community

The selection patterns that drive the bacterial community structuring in UCCs were inferred and illustrated through co-occurrence networks. Indeed, within a community, different members can experience common selection due to specific environmental factors without any actual direct or indirect interactions between them (Li et al. 2023). Here, considering the selection mediated by cultivation conditions, physical association, and cyanobacterial host, we evaluate the presence of different selection patterns, including (i) co-presences related to similar abundance patterns and (ii) mutual exclusions related to opposite abundance patterns (rare species vs. abundant species) (Vázquez et al. 2007). Thus, when highly significant differences in abundance are present across community members—as in the case of UCCs dominated by cyanobacteria hosts in terms of relative abundance (Supplementary Fig. S2)—exclusion patterns are expected. For this reason, we applied the same methodology to compute two types of co-occurrence networks: the first considers all the members of the UCCs that include the dominant cyanobacteria (i.e. the host) and associated heterotrophic bacteria, while the second excludes the dominant cyanobacteria OTUs, evaluating only the interactions among the associated heterotrophic bacteria (relative abundance distribution in Supplementary Fig. S2). The CoNet plugin (Faust and Raes 2016) in Cytoscape (Shannon et al. 2003) was used to identify positive (co-presence) and negative (mutual exclusion) selection patterns among OTUs using Spearman’s correlation coefficient (*ρ* > |0.5| and *P* < .01) to provide information on bacterial taxa that may respond robustly and similarly/differentially to environmental conditions (i.e. cultivation and UCC-host). Edge-specific permutation and bootstrap score distributions were performed with 100 iterations. The co-occurrence network was visualized with Gephi (Bastian et al. 2009), and default network parameters were used to describe their topology.

### Isolation, purification, and identification of associated heterotrophic bacteria

Aliquots from UCCs were plated on tryptic soy agar (TSA) and BG11. The plates were incubated at 30°C for up to 2 weeks. Colonies of different morphotypes were picked, purified, and preserved in 25% glycerol at −20°C. Genomic DNA from 24 isolates was extracted according to Phenol–Chloroform extraction method (McKiernan and Danielson 2017). PCR amplification of 16S rRNA gene was done using universal primers 27F (Edwards et al. 1989) and 1492R (Weisburg et al. 1991). The amplified DNA fragments were sequenced and compared with the nucleotide sequences available in NCBI (http://www.ncbi.nlm.nih.gov/) using the BLAST (Basic Local Alignment Search Tool) program.

## Results

### Characterization of the bacterial community associated with desert spring microbial mats

Microbial mats collected from the hydrothermal pool of Ksar Ghilane (Fig. 1B–G) present mixed microbial assemblages with macroscopically visible agglomerates containing cyanobacteria cells. Different morphospecies occurred within the green layer of the microbial mat (Fig. 1H–L, Supplementary Fig. S3). Specifically, thin, filamentous cyanobacteria were particularly prevalent, characterized by solitary or agglomerated filaments that appeared straight or slightly undulating without any evidence of branching. Filaments with helical shapes (spirulina-like) and diatoms were also observed, indicating the complexity of these benthonic microbial mats. Amplicon sequencing confirmed the presence of various cyanobacterial taxa (9 OTUs; Supplementary Data S2A). The helical-shape *Arthrospira* (OTU46) was consistently detected in all the microbial mat samples (relative abundance, 4%–21%), while OTUs belonging to an unclassified cyanobacteria (OTU29), *Microcystis* (OTU43), and *Geitlerinema* (OTU36) were relatively high abundant (up to 47%) but differently distributed across samples. The other cyanobacterial OTUs identified as unclassified *Oxyphotobacteria* (OTU37), *Leptolyngbya* (OTU1), *Nodosilinea* (OTU2 and OTU416), and *Arthronema* (OTU3) were detected with low relative abundance. Along with cyanobacteria, 38 bacterial OTUs were found, belonging mainly to *Actinotalea, Aeromonas, Ahniella, Agromyces, Brevundimonas, Fontimonas, Hydrogenophaga, Porphyrobacter, Pseudoxanthomonas, Salinarimonas, Terrimonas*, and *Zhizhongheella* (Supplementary Data S2A). However, only 13 of these bacterial OTUs were present in all the tree microbial mat samples.

### Establishment of UCCs from spring microbial mats

**Figure 2.**
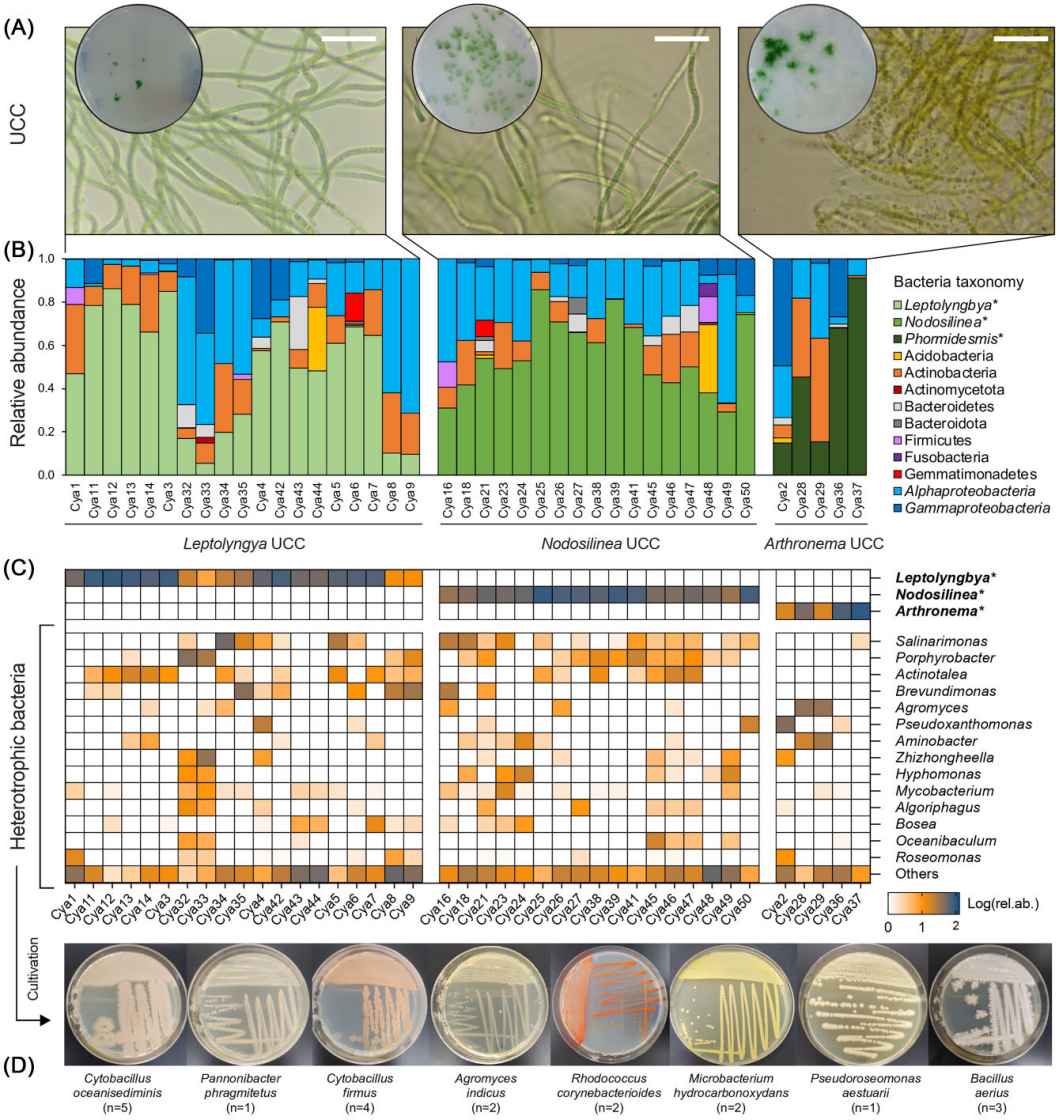
(A) Representative image of UCC plates and cyanobacteria at the optical microscope. Microscope images of the 41 UCCs are reported in Supplementary Fig. S5. Relative abundance at (B) phylum/class and (C) genus levels of bacterial community detected in the 41 UCCs; cyanobacteria are marked with a star and always reported at the genus level. All the remaining genera and non-classified genera were merged and presented as ‘Others’. (D) Example of morphological diversity of bacteria isolated from UCCs on TSA and BG11. Taxonomic affiliations are also reported at the species level. The full collection is presented in Supplementary Table S3.

Portions of the spring microbial mat were cultured for 60 days to promote the enrichment and growth of cyanobacteria. During this period, distinct tuft morphologies developed (Supplementary Fig. S4), and 11 primary cultures were selected based on pigmentation, gas production, and floating capacity (Supplementary Table S1). These cultures were then purified following solid–liquid alternate cultivation (see the ‘Materials and Methods’ section), and 50 sub-cultures were obtained. Among them, 41 UCCs were characterized by the presence of only one cell morphotype/OTU of cyanobacteria (Fig. 2A, Supplementary Fig. S5), while nine cultures were discarded because they contained multiple morphologies/OTUs or cyanobacteria did not develop (Supplementary Data S2B and C). Amplicon sequencing analysis showed that cyanobacteria were the most abundant taxonomic group across the UCCs, represented by four OTUs, with relative abundances ranging from 5.6% to 91.3% (Fig. 2B, Supplementary Data S2B). The cyanobacteria OTUs were classified into three genera: *Leptolyngbya, Nodosilinea*, and *Arthronema* (Supplementary Data S1 and S2B). Based on this, the 41 UCCs were divided into three groups: the first group consisted of 19 UCCs that contained one OTU of *Leptolyngbya* (LeUCC), the second group was composed of 17 UCCs that contained one or two OTUs of *Nodosilinea* (NoUCC), and the third group was formed by five UCCs with one OTU of *Arthronema* (ArUCC). In the UCCs, the relative abundance of the forming cyanobacteria varied, ranging from 6% to 86% for *Leptolyngbya*, from 32% to 86% for *Nodosilinea* (both OTUs), and from 15% to 91% for *Arthronema*. Notably, the cyanobacterial community in the original microbial mat exhibited greater cyanobacterial diversity compared to the UCCs, with the cyanobacteria that became enriched in the UCCs initially present at very low relative abundances (Supplementary Data S2A). This suggests that the cyanobacteria forming the UCCs underwent significant proliferation and/or were preferentially selected by the cultivation conditions we used.

### Structure and diversity of UCCs’ bacterial communities

**Figure 3.**
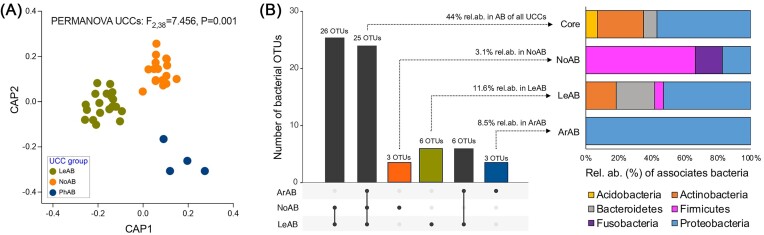
(A) Canonical analysis of principal coordinates and PERMANOVA results based on Bray–Curtis. UCCs belonging to the three groups are indicated in different colours. (B) Upset plot showing the number of OTUs present in the UCC core and those specific to each UCC with their relative abundance. LeAB: *Leptolyngbya-*associated bacteria, NoAB: *Nodosilinea-*associated bacteria, and ArAB: *Arthronema-*associated bacteria. Taxonomic affiliation of core and UCC-specific OTUs non-belonging to cyanobacteria is also reported at the phylum level. Details in Supplementary Table S2.

We examined the similarity among the structure of bacterial communities associated with the UCCs groups (beta-diversity) by applying a constrained ordination analysis to the Bray–Curtis similarity distance matrix (Fig. 3A). The results reveal that the three UCC groups harbour significantly distinct bacterial communities (PERMDISP: *F*_2.38_ = 0.077, *P* = .565; PERMANOVA: *F*_2.38_ = 7.456, *P* = .001; pairwise comparisons, *P* < .001), all of which were different from the originating-microbial mat communities (Supplementary Fig. S6). The number of observed OTUs (richness) differed between NoUCC and LeUCC, with ArUCC displaying intermediate values (ANOVA UCCs: *F*_2.38_ = 8.017, *P* = .0012; comparisons in Table 2). In contrast, no significant differences were found for the Shannon index across UCCs (ANOVA UCCs: *F*_2.38_ = 1.22, *P* = .27). Overall, the UCCs’ richness values were lower than those measured in the former microbial mats, though the Shannon index did not show significant variation (Table 2).

**Table 2.** Alpha diversity of spring microbial mat samples and UCCs was reported as richness (observed OTUs) and Shannon index.

Alphadiversity	Microbial mat	ArUCC	LeUCC	NoUCC	ANOVA (*F, df*, and *P*)
Number of samples	3	5	19	17	
Richness	25.0 ± 3.0(a)	11.6 ± 3.4(bc)	11.0 ± 3.8(b)	16.6 ± 5.0(c)	*F*_3.40_ = 12.41, *P* < .0001
Shannon	2.11 ± 0.22	1.23 ± 0.64	1.34 ± 0.55	1.54 ± 0.46	*F*_3.40_ = 2.11, *P* = .0569

LeUCC: *Leptolyngbya*-associated bacteria; NoUCC: *Nodosilinea*-associated bacteria; ArUCC: *Arthronema*-associated bacteria. Data are reported as average and standard deviation; the number of samples analysed is also reported for each group. Ordination one-way ANOVA results are reported, along with letters indicating shared significance groups (Tukey’s multiple comparisons test).

The bacterial composition of the 41 non-xenic UCCs was further investigated, considering only the associated heterotrophic bacteria, i.e. excluding the cyanobacteria hosts. We detected 68 bacterial OTUs classified into 7 phyla, 10 classes, 26 orders, 41 families, and 50 genera (Supplementary Data S2B). The distribution and prevalence of such bacteria were significantly affected by the niche partitioning mediated by cyanobacteria monoculture (Figs 2B, C and 3A). Indeed, besides we observed a great level of heterogeneity among UCC replicates (Fig. 2B and C), the bacterial communities’ diversity (OTU level) was consistently influenced by the UCC group (PERMDISP: *F*_2.38_=2.97, *P* = .147; PERMANOVA: *F*_2.38_=3.693, *P <* .006; pairwise comparisons, *P* < .01; Supplementary Fig. S7), suggesting that while the overall community structure may still vary due to external factors (e.g. random colonization events across replicates), cyanobacterial hosts drive the assembly of associated bacteria under laboratory conditions. For instance, *Alphaproteobacteria* consistently dominated the UCC communities but with differential distribution across UCCs (17.5 ± 2.9%, 24.3 ± 5.4%, and 25.6 ± 3.2% of relative abundance in ArUCC, LeUCC, and NoUCC communities, respectively) and among replicates in each group (Fig. 2B). This is also the case of *Gammaproteobacteria* and *Actinobacteria*, which ranged from 2.4 ± 1% to 15.1 ± 5% and from 7.9 ± 1.6% to 16.5 ± 4.5%, respectively (Fig. 2B). As well, we detected several common OTUs, i.e. UCC-core formed by OTUs present at least in one UCC replicate across the three groups. The UCC-core consisted of 25 bacterial OTUs, representing 44% of the total relative abundance of the heterotrophic bacterial portion (Fig. 3B, Supplementary Table S2). Dominant members of this core community include *Alphaproteobacteria* as *Salinarimonas* (6.1%), *Pseudoxanthomonas* (4.1%), and *Aminobacter* (3.9%), *Actinobacteria* of the *Agromyces* and *Mycobacterium* genera (4.5% and 2.3%), and *Gammaproteobacteria* of the *Zhizhongheella* genus (3.7%) (Fig. 3B, Supplementary Table S2). We also identified, even if with a minor entity, UCC-specific OTUs for each group; three OTUs accounting for 9% and 3% of the relative abundance were specific for ArAB and NoAB, respectively, and six OTUs accounting for 12% of the relative abundance were specific for NoAB (Fig. 3B). The ArAB-specific bacteria were composed by *Alphaproteobacteria* (*Skermanella* and *Rhizobium* genera), while those specific of NoAB were dominated by *Bacilli*, such as *Bacillus* and *Streptococcus*; the composition of LeAB-specific bacteria was more variegate, encompassing several phyla/classes, such as the Proteobacteria (i.e. *Brevundimonas* and *Haemophilus* genera), *Actinobacteria* (*Dietzia* and *Aeromicrobium*), *Bacilli* (*Bacillus*), and *Bacteroidia* (Flavihumibacter) (Fig. 3B, Supplementary Table S2). The remaining taxonomic groups are scattered among UCCs, such as the case of *Bacillii* and *Gemmatimonadetes*, which were only detected in LeAB and NoAB, and *Saccharimonadia* in NoAB.

### Cultivation of heterotrophic bacteria associated with the UCCs

Bacterial diversity associated with UCCs was also explored by characterizing the cultivable portion of heterotrophic bacteria. A total of 24 isolates were obtained using TSA medium and BG11 agar medium (Supplementary Table S3). The isolate’s morphological traits, such as size and colour, reveal a high level of variation (Fig. 2D). The BLAST search in NCBI based on the 16S rRNA sequences showed that the bacterial isolates belong to ten species within six genera, including nine *Cytobacillus* spp., four *Bacillus* sp., four *Microbacterium* spp., three *Agromyces* spp., two *Rhodococcus* sp., one *Pseudoroseomonas* sp., and one *Pannonibacter* sp. (Supplementary Table S3). Four were obtained from LeUCC (i.e. *C. oceanisediminis, R. corynebacterioides, M. hydrocarbonoxydans*, and *Ps. a estuarii*), three from *Nodosilinea* UCC (*Pa. phragmitetus, A. indicus*, and *A. arachidis*), two from both LeUCC and NoUCC (*B. aerius* and *C. firmus*), and one from ArUCC (*M. marinum*) (Supplementary Table S4).

To assess the prevalence of isolates within the overall community (defined by amplicon sequencing), the 16S rRNA gene sequences of the 24 bacterial isolates were compared with the bacterial OTUs (Supplementary Data S3). Only six isolates showed more than 97% identity with at least one OTU: *A. indicus* K7811, K1431, and K1432 strains were related to OTU9, *P. phragmitetus* K7311 to OTU62, *M. hydrocarbonoxydans* K1240 to OTU11, and *P. aestuarii* to OTU44 (Supplementary Data S3). These OTUs represented 31.5%, 2.1%, and 6.3% of the heterotrophic bacterial communities of ArUCC, LeUCC, and NoUCC (excluding reads of cyanobacteria). Indeed, the analysis revealed that although a bacterial strain was obtained only from a UCC group, the associated OTU was detected across multiple UCCs (Supplementary Table S5). For instance, *Microbacterium* was isolated from LeUCC, but the OTU11 was found across all the UCCs, even with higher relative abundance than the UCC source (1.5%, <1%, and 4.2% in ArAB, LeAB, and NoAB communities, respectively). Yet, *Agromycetes* strains were isolated from ArUCC and NoUCC, but the related OTU9 was also detected in LeUCC (<1%). Similarly, the *Pseudoroseomonas* strain was isolated from LeUCC, but the associated OTU44 had a higher relative abundance in ArUCC (1.3% vs. 3.2%, respectively).

### Variation in bacterial co-occurrence network topology across UCCs

**Figure 4.**
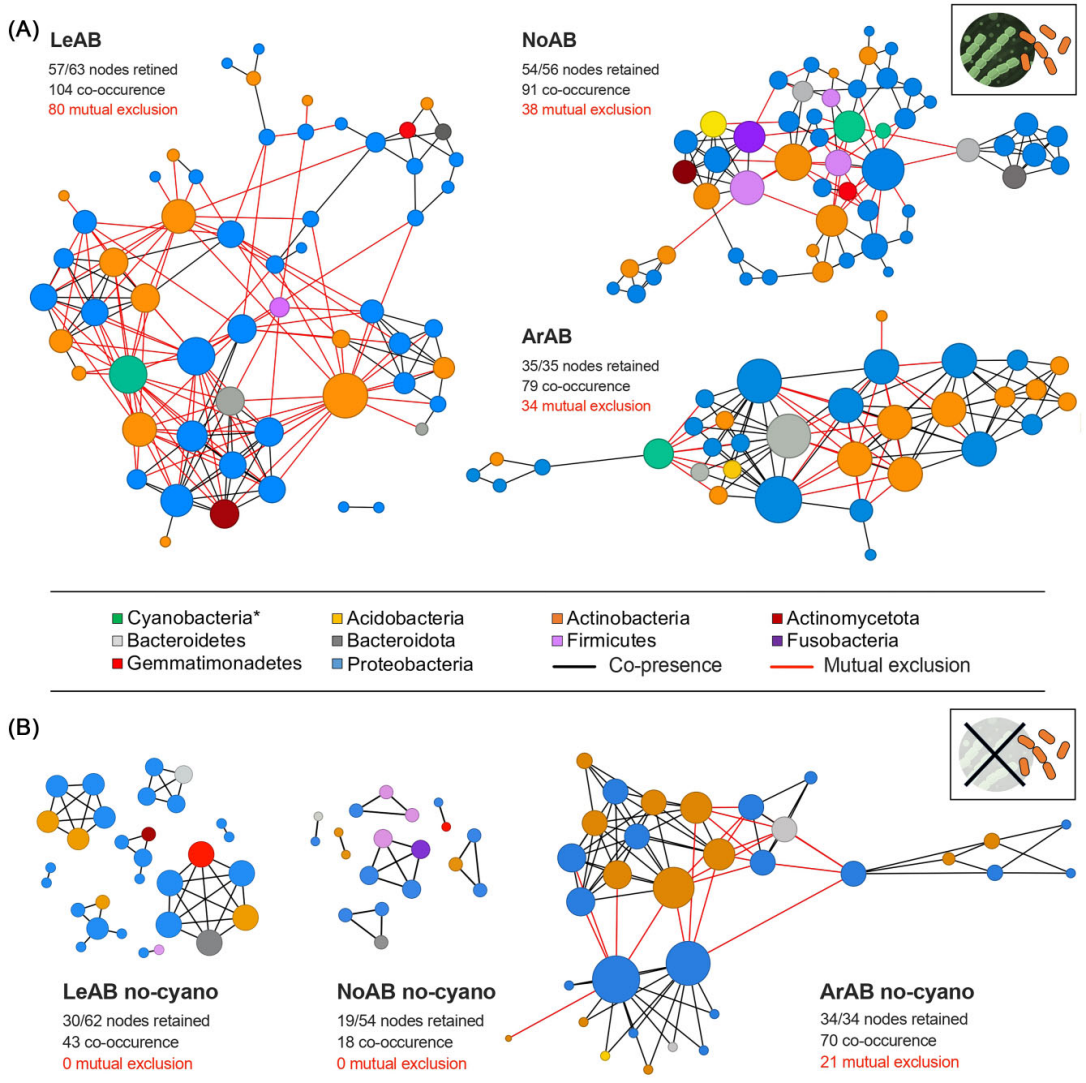
Co-occurrence network analysis of (A) the entire bacterial communities, including the cyanobacteria OTUs, within the three UCCs, LeAB, NoAB, and ArAB. (B) Removal of cyanobacteria OTUs (i.e. the hosts) to evaluate the co-occurrence network of the heterotrophic bacteria within the UCCs. Each node represents different bacteria (OTUs), and each edge represents significant selection patterns that can be classified as positive (co-occurrence) or negative (exclusion). Node size indicates the degree of connection (edge) of each OTU, whereas the colours indicate its taxonomic affiliation. The number of nodes and edges is reported for each network.

To assess common outcomes of population selection in the bacterial community associated with the three UCC groups, we performed different co-occurrence networks. In the first analysis, the host (cyanobacteria) and associated heterotrophic bacteria were both retained (Fig. 4A). The networks of LeAB, NoAB, and ArAB were composed of 57, 54, and 35 nodes, respectively, interconnected by 184, 129, and 113 edges. All networks were tightly connected with limited modularity, moderate cluster coefficient, and short path length, but all had low density (Supplementary Table S6). Independent of the UCC network, mutual exclusion patterns were mainly observed between cyanobacteria and the associated heterotrophic bacteria, even if they were also present within and between heterotrophic bacterial phyla. Degrees (number of links of a node) showed that the nodes with the higher number of connections belong to the cyanobacteria hosts (the most abundant component of the UCCs) and to Proteobacteria and Actinobacteria that dominate the heterotrophic bacteria. However, each UCC group presented a unique node composition (Fig. 4A) that reflects the unicity of each community. For example, Firmicutes and Fusobacteria were keystone nodes in NoAB, while a Bacteroidetes-OTU had this function in ArAB.

A second set of co-occurrence networks was computed by excluding the host (dominant OTU) to focus only on the co-occurrence patterns among heterotrophic bacteria (Fig. 4B). This approach was necessary because co-occurrence models, like CoNet, infer correlations based on relative abundance, and dominant host OTUs (Supplementary Fig. S2) can lead to inflated or misleading patterns of selection (Vázquez et al. 2007). For instance, by removing the dominant host, the links among the bacteria were reduced, with far fewer members of the community retained in the LeAB and NoAB co-occurrence networks (Fig. 4B). While the ArAB co-occurrence network did not show major differences in terms of links and topology, those of LeAB and NoAB were more fragmented, with several isolated modules dominated by co-occurrence patterns (Fig. 4B, Supplementary Table S7). These differences highlight how the imposed segregation of the heterotrophic-associated populations influenced the structure and complexity of the LeAB and NoAB co-occurrence networks, suggesting the presence of differential patterns of imposed selection in the presence/absence of the host (Fig. 4B).

## Discussion

Ecological interactions between Cyanobacteria and heterotrophic bacteria are crucial for the structure and functioning of photosynthetic microbial mats in hydrothermal sources (Grossart et al. 2006, Azam and Malfatti 2007, Rabus et al. 2015). However, the complexity of these interactions makes it challenging to isolate and define the role of each community member. By transitioning to simplified cultures, such as UCCs, we can more effectively study the influence of photosynthetic organisms like cyanobacteria on the recruitment and assembly of heterotrophic bacterial communities. Within this framework, this study encompassed the isolation and taxonomic identification of UCCs derived from the microbial mat of the desert hydrothermal pool of Ksar Ghilane at the northwest edge of the Grand Erg Oriental in the Sahara Desert—one of the several Tunisian geothermal springs that still have received little attention (Sayeh et al. 2010).

Standard techniques of cyanobacterial isolation aim to minimize the number of microorganisms while maintaining the integrity of those closely attached to the cyanobacterial sheaths (Halary et al. 2022). These methods are designed to retain ecologically important heterotrophic bacteria that form symbiotic relationships with cyanobacteria, as they often play critical roles in nutrient cycling, organic matter decomposition, and community stability. However, the possibility of obtaining pure-cyanobacterial culture is further complicated by the polymorphism and phenotypic plasticity of these bacteria (Neilan et al. 1995, Casamatta et al. 2002), making their identification difficult. For instance, it has been shown that cyanobacteria change their morphology in response to environmental factors, such as nutrient availability (Ghadouani and Pinel-Alloul 2002, Dvornyk and Nevo 2003, Bittencourt-Oliveira et al. 2009, Whitman and Agrawal 2009). Changes in cell size and shape contribute to buoyancy regulation, allowing cyanobacteria to be in an optimum position in the water to exploit and search for light and nutrients (Sengupta et al. 2022, Borics et al. 2023). Despite these challenges, after multiple passages of decimal dilution, we obtained 41 UCCs derived from the desert spring microbial mat of Ksar Ghilane. This process allowed the cultivation of three filamentous cyanobacteria, identified as *Leptolyngbya, Nodosilinea*, and *Arthronema*. However, these cyanobacteria were barely detectable in the spring microbial mats used as the initial inoculum that contained several more cyanobacteria, including helical-shaped *Arthrospira, Microcystis*, and *Geitlerinema*. This could be explained by the isolation methods and cultivation conditions used that did not favour the growth of such other cyanobacteria and/or preferentially select the proliferation of the taxonomic groups obtained (Gaget et al. 2017).

Despite *Leptolyngbya, Nodosilinea*, and *Arthronema* not being dominant in the samples of microbial mats, these cyanobacteria have been found in several thermal springs worldwide. *Leptolyngbya*, the most predominant genus detected among our UCC, is known to be widespread in hot springs and various other aquatic habitats worldwide, where it contributes to the formation of sedimentary rocks (among others, stromatolite and travertine formations) via calcium carbonate precipitation (Komárek 2003, Dadheech et al. 2013, Amarouche Yala et al. 2014, Kanellopoulos et al. 2022, Vahrenkamp et al. 2024). The second most abundant cyanobacterium belongs to the genus *Nodosilinea*. Members of this genus inhabit diverse ecosystems, ranging from extreme environments like the McMurdo Dry Valleys (Costa et al. 2016) and thermal springs (Heidari et al. 2018) to marine environments (Li and Brand 2007) and freshwater ecosystems (Perkerson III et al. 2011). A few OTUs were assigned to *Arthronema*, isolated for the first time from the Asian deserts in Kuwait and Nepal (Komárek and Lukavský 1988). This cyanobacterium thrives in extreme habitats like deserts, resisting temperature and light variations (Iliev et al. 2006). The growth dynamics, pigment production, and antioxidant enzyme activity of *A. africanum* are impacted by its adaptive stress responses to heavy metal exposure, which define its ecology (Karcheva et al. 2022). Besides, studies demonstrated that *A. africanium* cells maintain a constant cell membrane fluidity by adjusting the fatty acids composition at various temperatures, as the percentage of linolenic acid in their cells decreased from 33% to 0.5% under *ex situ* conditions. Although cyanobacteria have demonstrated remarkable adaptability and resilience, fulfilling important ecological roles in hydrothermal ecosystems (Dabravolski and Isayenkov 2022), we speculate that the overall reduced diversity observed in the Ksar Ghilane pool (9 OTUs, dominated by *Arthrospira, Microcystis*, and *Geitlerinema*) can be attributed to the overall climatic condition and isolation of such desert oasis, as well as to the water quality, rather than other geological factors. For instance, high levels of aluminium were detected in this water source (Table 1). Aluminium is a metal known to be toxic for cyanobacteria in a dose- and species-specific manner (Hamed et al. 2019); it can inhibit photosynthesis, induce oxidative damage (Pradhan et al. 2020), and enhance cell lysis (Guimarães Neto et al. 2019). Dawah and co-workers (2015) showed that the number of blue-green algae in the presence of aluminium was 98% less than in control samples.

Cyanobacteria are also supported by diverse associated microorganisms, including cocci and bacilli, that colonize the cyanosphere of these photosynthetic microorganisms (Pérez-Carrascal et al. 2021, Halary et al. 2022). This association and the establishment of complex microbial consortia are pivotal for the stability and fitness of the latest in both natural environments and laboratory conditions (Morris et al. 2008, Jackrel et al. 2021, Halary et al. 2022). Presumably, such associations are driven by the photoautotrophic metabolism of cyanobacteria, which, by exchanging and recycling organic matter and key nutrients, significantly impacts the metabolic context of the cyanosphere and, thus, the composition of the associated bacterial community (Parveen et al. 2013, Louati et al. 2015, Cook et al. 2020, Zheng et al. 2020). Indeed, cyanobacteria differ in different ways regarding their respective sheath and exudate production, affecting the selection/recruitment patterns. Specifically, our study detected 68 heterotrophic bacterial OTUs and obtained 24 isolates across the three UCC groups. Considering the methods applied for the enrichment and isolation of UCCs and the results obtained by previous studies (Cole et al. 2014), these bacteria can be considered an integral component of the cyanosphere rather than contaminants of the culture medium. In particular, we found that the heterotrophic bacterial components were differentially distributed across the UCCs, defining the assembly of different communities. While there was a certain level of heterogeneity across the different cultures within each UCC group, their diversity and assembly are consistently influenced by the cyanobacterial taxa forming them. The observed heterogeneity does not necessarily imply that cyanobacteria have no influence; instead, it highlights that the mechanisms regulating such association are complex and not solely deterministic. Cyanobacteria can mediate the association with specific bacterial taxa, but the overall community structure can still vary due to external factors, such as random colonization events or stochastic assembly during culture establishment.

The heterotrophic bacterial core of our UCCs is predominantly composed of Proteobacteria and Actinobacteria, followed by Bacteroidetes and Acidobacteria, even if their prevalence is cyanobacteria genus-specific. Proteobacteria have been reported in hot springs at various locations (Tekere et al. 2011, Pagaling et al. 2012, Chan et al. 2015, Ghilamicael et al. 2017), owing to their thermophilic nature, which allows them to adapt to extreme environments (Ward et al. 1998, Keshari et al. 2022). Genetic analyses also revealed that many genes found in cyanobacteria share homology with those of Proteobacteria (Timmis et al. 2004, Encinas et al. 2014), as in the case of the high homology shared between carboxysomes encoded by certain species of cyanobacteria and Proteobacteria (Rae et al. 2013). Also, Actinobacteria, ubiquitous in several edaphic and aquatic habitats (Lauber et al. 2009, Jiang et al. 2012, Zhang et al. 2014, Yang et al. 2015), have been shown to form specific associations with cyanobacteria (Woodhouse et al. 2016), as in the case of *Rhodoluna lacicola* playing essential roles in nutrient cycling (Eckert et al. 2012, Ghai et al. 2014) and those actinobacteria species that, by degrading cyanobacterial toxins, can favour the fitness of the host (Mou et al. 2013). Along with the core bacteria, each UCC hosts specific heterotrophic bacteria, including *Alphaproteobacteria* (*Brevundimonas, Skermanella*, and *Rhizobium*), *Gammaproteobacteria* (*Haemophilus*), *Bacilli* (*Bacillus* and *Streptococcus*), *Actinobacteria* (*Aeromicrobium* and *Dietzia*), *Bacteroidiia* (*Flavihumibacter*), and *Fusobacteriia* (*Fusobacterium*). Similar studies have shown that such environmental filtering, driven by dominant phototrophs, often selects specific heterotrophic bacteria based on nutrient exchange and niche complementarity (Moraes et al. 2016, Gao et al. 2020, Cobos et al. 2022, Kai et al. 2023). In our work, we also purified some of the heterotrophic bacteria associated with the different UCCs, including members of the *Agromyces, Cytobacillus, Bacillus, Microbacterium, Pannonibacter, Pseudoroseomonas*, and *Rhodococcus* genera. While some of them were isolated from specific UCCs, a comparison with the OTUs dataset revealed that many isolates can be detected across all communities even if in low abundance part of the ‘rare’ component of the community (Chandarana et al. 2023, Mitter et al. 2023). The bacterial species cultivated in our study have previously been described as both symbionts and pathogens of other cyanobacteria species. For instance, *Microbacterium* isolated from the mucilaginous sheath of *Microcystis aeruginosa* solubilises both inorganic and organic phosphate, enhancing the growth of this cyanobacteria (Zhang et al. 2017). In contrast, several *Bacillus* have been reported to inhibit the growth and cause lysis of cyanobacteria, particularly across *Anabaena* species (Gao et al. 2023). The relative specificity and relatively limited diversity of bacterial communities associated with UCCs imply strong selective pressure exerted by biotic (cyanosphere) and abiotic factors (temperature, medium) (Buchan et al. 2014), a typical pattern observed in microbial communities inhabiting specialized niches (Mathur et al. 2007, Mayerhofer et al. 2021). By computing UCCs’ co-occurrence network, we observed high connectivity of the overall community (cohesive network), with more co-occurrence patterns than mutual exclusions. It indicates that the bacterial species have/share similar niches (Faust and Raes 2012), as in the case of heterotrophic bacteria associated with the cyanosphere. Bacteria can indeed benefit from the production and accumulation of substrates released by cyanobacteria, contributing in turn to essential nutrients like nitrogen or phosphorus through mineralization processes and their recycling (Cai et al. 2014, Xu et al. 2018). Besides such general outcomes, the specific dynamics will depend on factors such as the types of cyanobacteria, the composition of exudates, and the surrounding environmental conditions (Xu et al. 2018). Removing the host from the networks revealed indeed different scenarios of interactions across the three UCC communities of heterotrophic bacteria. While the heterotrophic bacteria in LeAB and NoAB form several small, non-connected modules, those in ArAB maintain a networking structure similar to that observed in the presence of the cyanobacteria host. The formation of modules and the absence of connection across these sub-communities indicate the presence of differential co-occurrence patterns that could be associated, for instance, with different niches (Faust and Raes 2012, Ratzke et al. 2020). Yet, the different patterns mediated by the presence/absence of cyanobacteria hosts in the network imply that the latter are pivotal in maintaining a structured community and cohesive microbial networking, possibly favouring the metabolic cooperation to maintain ecological functions as well as enhance survival and resilience of the metaorganism. Although the distribution of microbial species in the spring microbial mat is more complex and influenced by numerous other factors (not tested here), the results obtained from the UCC can be viewed as an estimation of the microbial responses to environmental filtering, where only a single cyanobacterium is present, allowing the identification and understanding of common selection patterns in the cyanobacteria-heterotrophic bacteria association.

## Conclusion

We demonstrated that uni-cyanobacterial consortia form complex communities where phototroph cyanobacteria maintain associations with distinct heterotrophic bacterial partners, even after several passages of dilution and isolation. The composition of these communities is primarily influenced by the cultivation conditions used. However, the taxonomy of the single cyanobacterium that took on the role of the primary autotrophic organism—specifically *Leptolyngbya, Nodosilinea*, and *Arthronema—*also plays a crucial role in shaping unique bacterial community assemblies. The different UCC communities form cohesive and tightly interconnected co-occurrence networks with similar topology, driven mainly by common selection patterns (i.e. cyanobacteria host and cultivation conditions). Our results showed how cyanobacteria mediate bacterial community assembly in non-axenic UCC, revealing the complexity of co-occurrence patterns occurring between autotrophs and heterotrophs in pure culture in laboratory conditions. Although these findings may not fully replicate the dynamics of more diverse natural systems (spring microbial mat), they provide valuable insights into the ecological processes and mechanisms that drive microbial community assembly, demonstrating the key roles cyanobacteria play in shaping their associated microbiomes. Future research could further explore these interactions by investigating additional environmental variables and testing for the role of specific metabolic exchanges between cyanobacteria and heterotrophs.

## Supplementary Material

fiae148_Supplemental_Files

## Data Availability

All raw sequencing data in FASTQ format are available in the NCBI Sequence Read Archive (SRA) BioProject database under accession number PRJNA1129164 as BioSamples SAMN42145605. The amplified 16S rRNA fragments were deposited in the GenBank under accession number PP958438.
