# Supplementary material for: Diversity and networking of uni-cyanobacterial cultures and associated heterotrophic bacteria from the benthic microbial mat of a desert hydrothermal spring
Source: FEMS Microbiol Ecol. 2024 Nov 18;100(12):fiae148. doi: 10.1093/femsec/fiae148 (PMC11650863; doi:10.1093/femsec/fiae148)
Supplement: fiae148_Supplemental_Files [file fiae148_supplemental_files.zip › Lassoued et al 2024_Supplementary Material.docx]

**Supplementary Material of**

**Diversity and networking of uni-cyanobacterial cultures and associated heterotrophic bacteria from the benthic microbial mat of a desert hydrothermal spring**

Khaoula Lassoued^1,2^, Mouna Mahjoubi^1^, Elias Asimakis^3^, Naima Bel Mokhtar^3^, Panagiota Stathopoulou^3^, Refka Ben Hamouda^1^, Olfa Bousselmi^1^, Ramona Marasco^4^, Ahmed Slaheddine Masmoudi^1^, Daniele Daffonchio^4^, George Tsiamis^3^ and Ameur Cherif^1*^

^1^BVBGR-LR11ES31, ISBST, Biotechpole Sidi Thabet, Univ. Manouba, Ariana 2020, Tunisia

^2^National Institute of Agronomy of Tunisia, University of Carthage, Tunis 1082, Tunisia

^3^Laboratory of Systems Microbiology and Applied Genomics, Department of Environmental Engineering, University of Patras, Agrinio 30100, Greece

^4^Biological and Environmental Sciences and Engineering Division (BESE), King Abdullah University of Science and Technology (KAUST), Thuwal 23955, Saudi Arabia

*Corresponding author: Ameur Cherif, e-mail: ameur.cherif@uma.tn; Tel./Fax: +216 70 527 882

**SUPPLEMENTARY FIGURES**

**Supplementary Figure S1.** Rarefaction curves are obtained from the 16S rRNA gene amplicon sequencing of the 41 UCCs. Samples are shown divided per UCC groups: *Leptolyngbya* UCC-associated bacteria*,* LeAB (n=19); *Nodosilinea* UCC-associated bacteria*,* NoAB (n=15); *Arthronema*UCC-associated bacteria, ArAB (n=5). In the x-axis is reported the sequencing depth and in the y-axis is the observed OUT (richness) per each sample. All samples have shown a Goods’ value of 100.

**Supplementary Figure S2.** Distribution of relative abundance of bacterial OTUs in the three UCC groups. Cyanobacteria OTUs are indicated in green, and those of heterotrophic bacteria in black.

**Supplementary Figure S3.** Visualisation of the spring microbial mats at the microscope.

**Supplementary Figure S4.** Cyanobacterial diversity within primary cultures obtained after 60 days of cultivation of spring microbial mats in BG11 medium (see Methods). Representative microscope images reveal different cyanobacterial morphologies, such as unicellular, colonial, and multicellular filamentous and spiral forms.

**Supplementary Figure S5.** Visualisation at the microscope of the 41 uni-cyanobacterial cultures (UCC). The UCCs were divided into three groups based on the morphological identification of the cyanobacteria enriched: (**A**) *Leptolyngbya* UCC, (**B**) *Nodosilinea* UCC and (**C**) *Arthronema* UCC.

(**A**) Representative microscope images of 19 *Leptolyngbya* UCCs.

(**B**) Representative microscope images of 15 *Nodosilinea* UCCs.

(**C**) Representative microscope images of 5 *Arthronema* UCCs.

**Supplementary Figure S6.** Comparison of the bacterial community associated with the microbial mat samples collected from the spring and UCCs obtained in the laboratory. (**A**) Canonical analysis of principal coordinates (CAPs) based on Bray–Curtis similarity matrix. (**B**) PERMANOVA multiple comparison results.


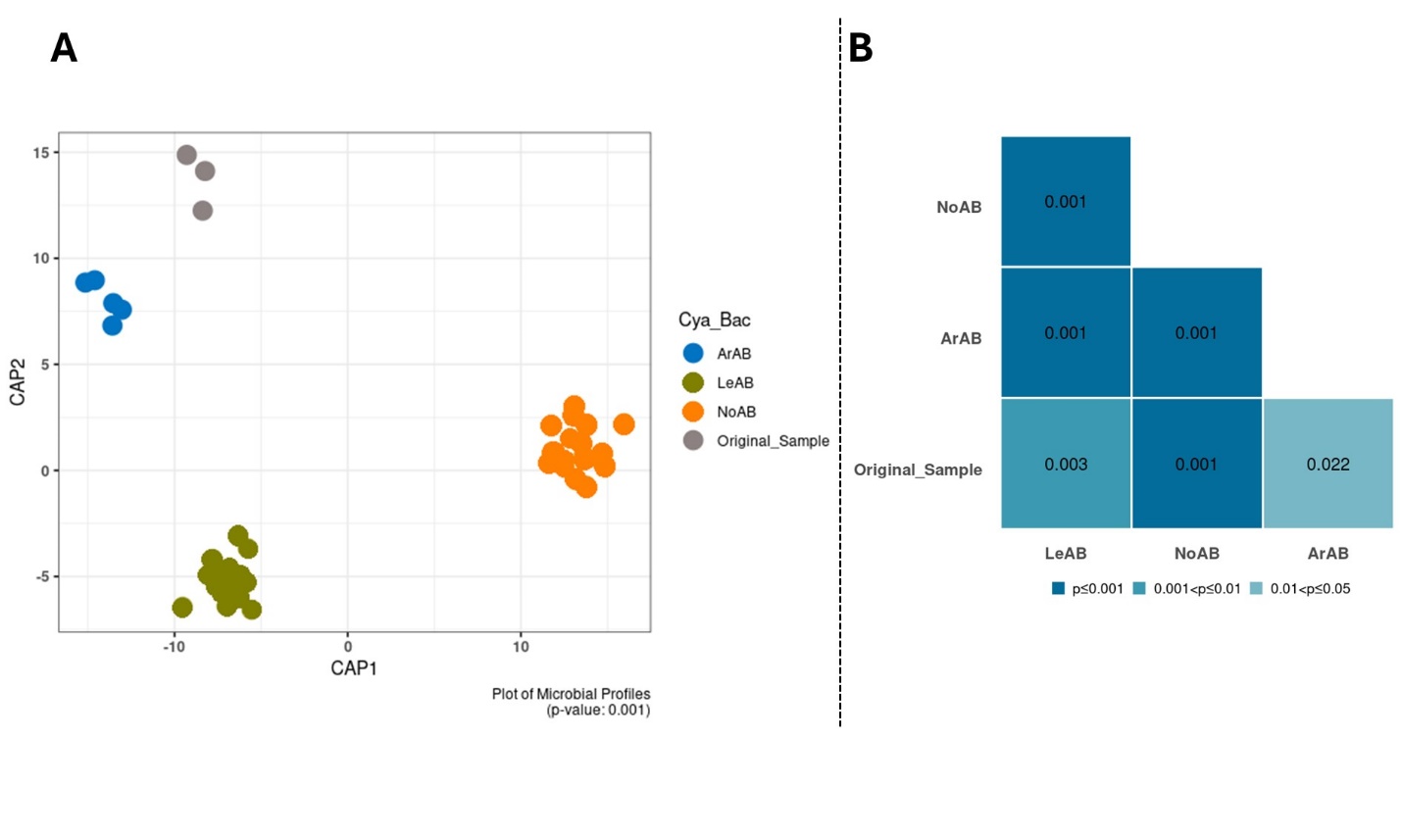


**Supplementary Figure S7.** (**A**) Canonical analysis of principal coordinates (CAPs) based on Bray–Curtis similarity matrix considering only OTUs of heterotrophic bacteria associated with Cyanobacteria. UCC groups are indicated with different colours. (**B**) PERMANOVA multiple comparison results.

**
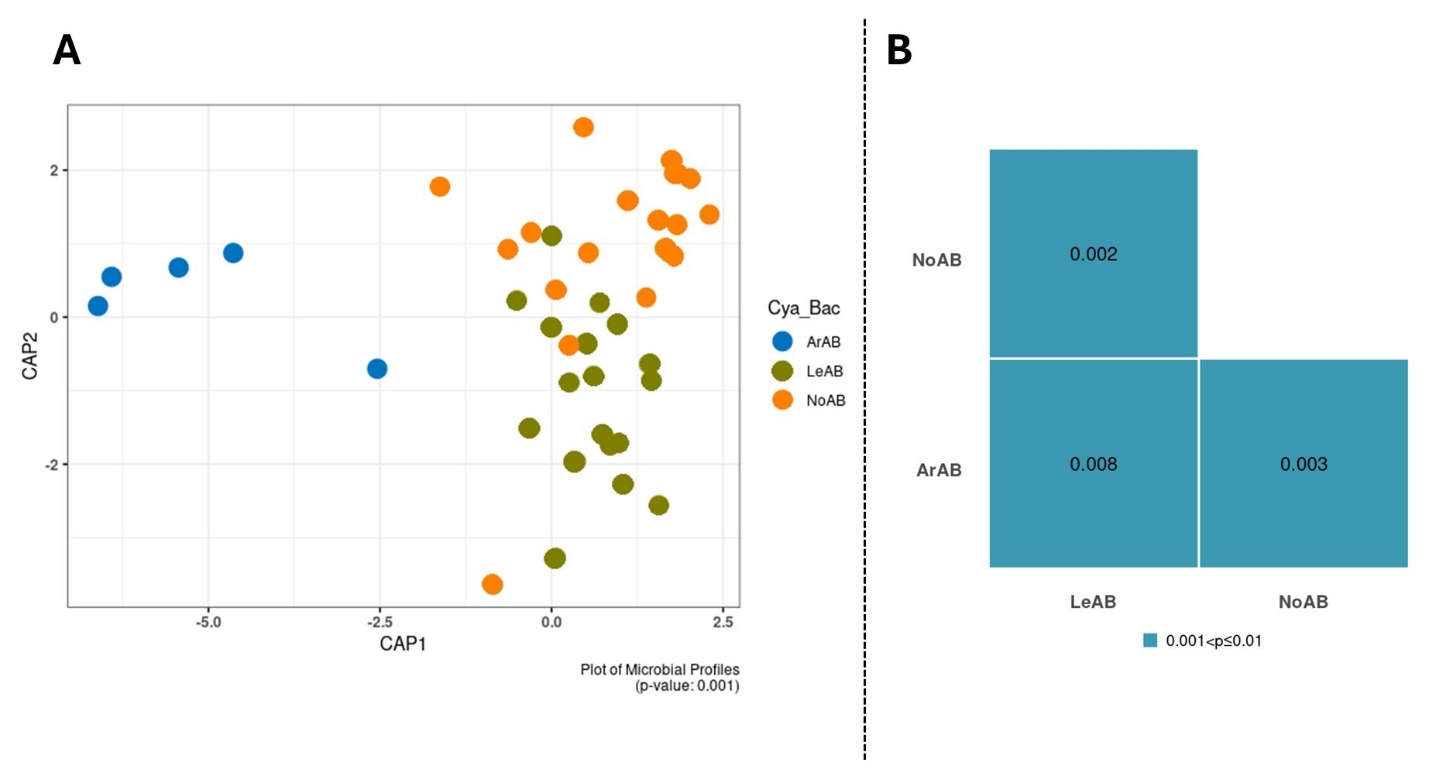
**

**SUPPLEMENTARY TABLES**

**Supplementary Table S1.** Morphological characterisation of the 11 main cyanobacterial cultures isolated from the Ksar Ghilane pool.

| Culture ID | Pigmentation | Gas vesicles | Floating capacity | Cyanobacterial cell morphology and structure |
| --- | --- | --- | --- | --- |
| PA1 | A spectrum of green tones characterized by the dominance of dark green pigment. | Yes | No | Aggregate to generate a concentrated biomass layer |
| PA2 | A spectrum of green tones characterized by the dominance of forest green pigment. | No | No | Vertical aggregation forming cone-structure  Aggregate to generate a concentrated biomass layer |
| PA3 | A spectrum of green tones characterized by the dominance of dark and light green pigments. | No | Yes | Vertical aggregation forming cone-structure  Aggregate to generate a compact biomass layer enveloped by intricate filamentous structures of fine complexity |
| PA4 | A spectrum of green tones characterized by the dominance of yellowish green pigments. | No | Yes | Vertical aggregation forming cone-structure  Aggregate to generate a compact biomass layer characterized by a mucilaginous appearance. |
| PA5 | A spectrum of green tones characterized by the dominance of forest green pigment. | No | Yes | Vertical aggregation forming cone-structure  Aggregate to generate a compact biomass layer enveloped by intricate filamentous structures of fine complexity |
| PA6 | A spectrum of green tones characterized by the dominance of forest green pigment. | Yes | Yes | Vertical aggregation forming cone-structure   - Aggregate to generate a compact biomass layer enveloped by intricate filamentous structures of fine complexity |
| PA7 | A spectrum of green tones characterized by the dominance of forest and yellowish green pigment. | Yes | Yes | Vertical aggregation forming cone-structure   - Aggregate to generate a compact biomass layer, characterized by a mucilaginous appearance and enveloped by intricate filamentous structures of fine complexity |
| PA8 | A spectrum of green tones characterized by the dominance of forest and yellowish green pigments. | No | Yes | - Aggregate to generate a compact biomass layer enveloped by intricate filamentous structures of fine complexity |
| PA9 | A spectrum of green tones characterized by the dominance of forest green pigment. | No | Yes | - Aggregate to generate small colonies and filamentous structures of fine complexity |
| PA10 | A spectrum of green tones characterized by the dominance of forest green pigment. | Yes | Yes | Vertical aggregation forming cone-structure   - Aggregate to generate a compact biomass layer enveloped by intricate filamentous structures of fine complexity |
| PA11 | A spectrum of green tones characterized by the dominance of forest and deep green pigments. | Yes | Yes | Vertical aggregation forming cone-structure   - Aggregate to generate a compact biomass layer, characterized by a mucilaginous appearance and enveloped y intricate filamentous structures of fine complexity |

**Supplementary Table S2.** Core OTUs present in the three UCC groups (at least in one of the UCC per group) and OTUs specific to each UCC group are listed. *Leptolyngbya-*UCC associated bacteria*,* LeAB; *Nodosilinea-*UCC associated bacteria*,* NoAB; *Arthronema-*UCC associated bacteria, ArAB.


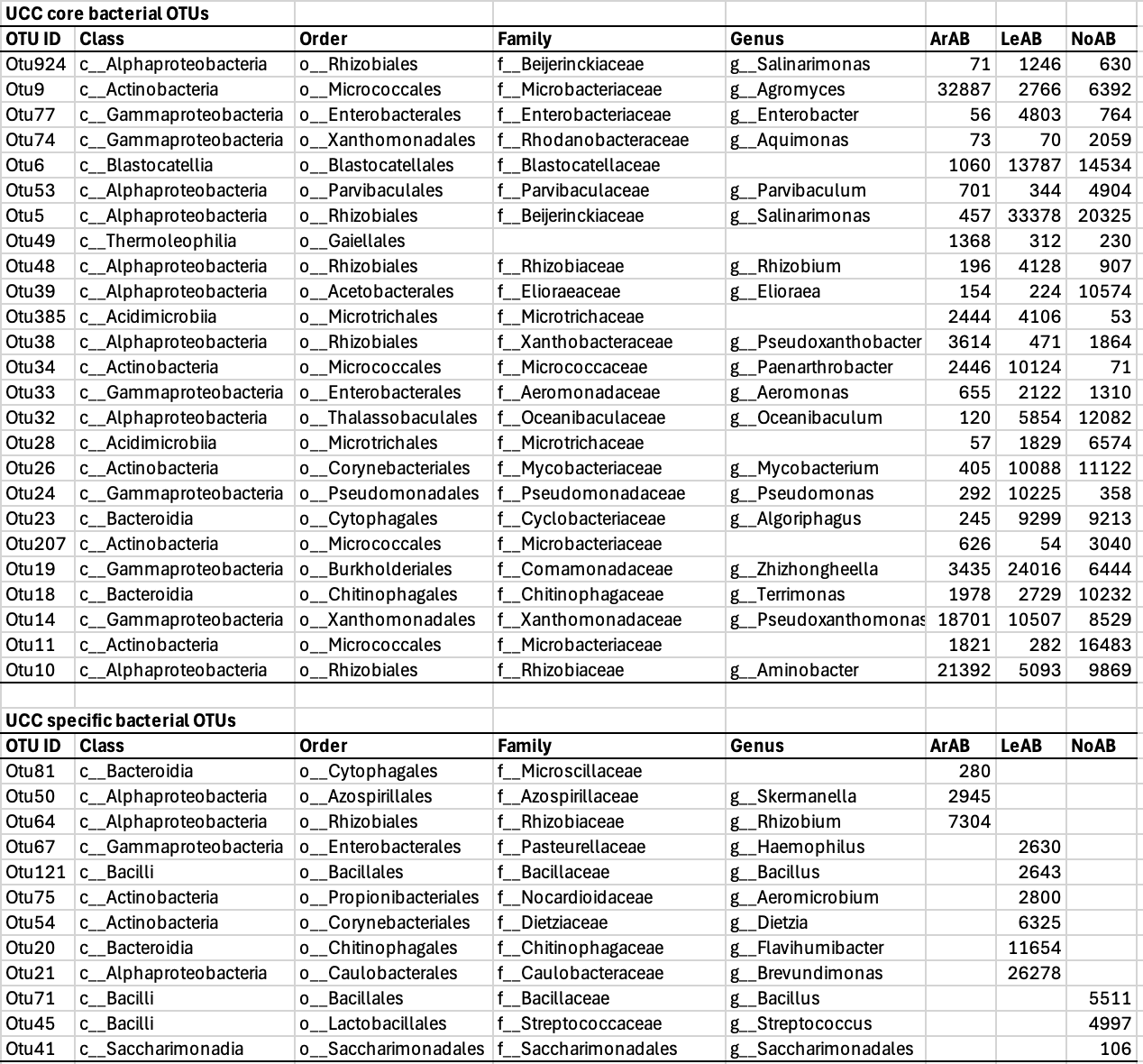


**Supplementary Table S3.** List of heterotrophic bacteria isolated from UCCs and their taxonomic identification based on sequencing similarity with the closest type strains.

| **Strain** | **Medium** | **Closest type strain** | **Taxonomy** |
| --- | --- | --- | --- |
| K1420 | TSA | *Cytobacillus oceanisediminis* H2 | Bacteria; Bacillota; Bacilli; Bacillales; *Bacillaceae; Cytobacillus.* |
| K1520 | TSA | *Rhodococcus corynebacterioides* DSM 20151 | Bacteria; Actinomycetota; Actinomycetes; Mycobacteriales; *Nocardiaceae; Rhodococcus.* |
| K1211 | BG11 | *Cytobacillus oceanisediminis* H2 | Bacteria; Bacillota; Bacilli; Bacillales; *Bacillaceae; Cytobacillus.* |
| K1220 | TSA | *Microbacterium hydrocarbonoxydans* BNP48 | Bacteria; Actinomycetota; Actinomycetes; Micrococcales; *Microbacteriaceae; Microbacterium.* |
| K1231 | TSA | *Cytobacillus oceanisediminis* H2 | Bacteria; Bacillota; Bacilli; Bacillales; *Bacillaceae; Cytobacillus.* |
| K1232 | TSA | *Cytobacillus oceanisediminis* H2 | Bacteria; Bacillota; Bacilli; Bacillales; *Bacillaceae; Cytobacillus.* |
| K1240 | TSA | *Microbacterium hydrocarbonoxydans* BNP48 | Bacteria; Actinomycetota; Actinomycetes; Micrococcales; *Microbacteriaceae; Microbacterium.* |
| K1450 | TSA | *Cytobacillus oceanisediminis* H2 | Bacteria; Bacillota; Bacilli; Bacillales; *Bacillaceae; Cytobacillus.* |
| K1521 | TSA | *Rhodococcus corynebacterioides* DSM 20151 | Bacteria; Actinomycetota; Actinomycetes; Mycobacteriales; *Nocardiaceae; Rhodococcus* |
| K1210 | TSA | *Pseudoroseomonas aestuarii* NBRC 105654 | Bacteria; Pseudomonadota; Alphaproteobacteria; Rhodospirillales; *Acetobacteraceae; Pseudoroseomonas* |
| K2400 | TSA | *Bacillus aerius* 24K | Bacteria; Bacillota; Bacilli; Bacillales; *Bacillaceae; Bacillus.* |
| K2131 | TSA | *Cytobacillus firmus*  NBRC 15306 | Bacteria; Bacillota; Bacilli; Bacillales; *Bacillaceae; Cytobacillus.* |
| K4100 | TSA | *Bacillus aerius*  24K | Bacteria; Bacillota; Bacilli; Bacillales; *Bacillaceae; Bacillus.* |
| K7311 | BG11 | *Pannonibacter phragmitetus*  C6-19 | Bacteria; Pseudomonadota; Alphaproteobacteria; Hyphomicrobiales; *Stappiaceae; Pannonibacter.* |
| K7411 | BG11 | *Cytobacillus firmus*  NBRC 15306 | Bacteria; Bacillota; Bacilli; Bacillales; *Bacillaceae; Cytobacillus.* |
| K7811 | BG11 | *Agromyces indicus* NIO-1018 | Bacteria; Actinomycetota; Actinomycetes; Micrococcales; *Microbacteriaceae; Agromyces.* |
| K1110 | TSA | *Microbacterium marinum*  H101 | Bacteria; Actinomycetota; Actinomycetes; Micrococcales; *Microbacteriaceae; Microbacterium.* |
| K1120 | TSA | *Microbacterium marinum*  H101 | Bacteria; Actinomycetota; Actinomycetes; Micrococcales; *Microbacteriaceae; Microbacterium.* |
| K1441 | TSA | *Cytobacillus firmus*  NBRC 15306 | Bacteria; Bacillota; Bacilli; Bacillales; *Bacillaceae; Cytobacillus.* |
| K1442 | TSA | *Cytobacillus firmus*  NBRC 15306 | Bacteria; Bacillota; Bacilli; Bacillales; *Bacillaceae; Cytobacillus.* |
| K1431 | TSA | *Agromyces indicus* NIO-1018 | Bacteria; Actinomycetota; Actinomycetes; Micrococcales; *Microbacteriaceae; Agromyces.* |
| K1432 | TSA | *Agromyces arachidis* AK-1 | Bacteria; Actinomycetota; Actinomycetes; Micrococcales; *Microbacteriaceae; Agromyces.* |
| K1610 | TSA | *Bacillus aerius* 24K | Bacteria; Bacillota; Bacilli; Bacillales; *Bacillaceae; Bacillus.* |
| K1621 | TSA | *Bacillus aerius* 24K | Bacteria; Bacillota; Bacilli; Bacillales; *Bacillaceae; Bacillus.* |

**Supplementary Table S4.** List of shared and specific bacterial isolates among UCCs. *Leptolyngbya-*UCC associated bacteria*,* LeAB; *Nodosilinea-*UCC associated bacteria*,* NoAB; *Arthronema-*UCC associated bacteria, ArAB.

| **Isolated bacteria** | **LeAB** | **NoAB** | **ArAB** |
| --- | --- | --- | --- |
| *Agromyces arachidis* | 0 | 1 | 0 |
| *Agromyces indicus* | 0 | 2 | 0 |
| *Bacillus aerius* | 1 | 3 | 0 |
| *Cytobacillus firmus* | 1 | 3 | 0 |
| *Cytobacillus oceanisediminis* | 5 | 0 | 0 |
| *Microbacterium hydrocarbonoxydans* | 2 | 0 | 0 |
| *Microbacterium marinum* | 0 | 0 | 2 |
| *Pannonibacter phragmitetus* | 0 | 1 | 0 |
| *Pseudoroseomonas aestuarii* | 1 | 0 | 0 |
| *Rhodococcus corynebacterioides* | 2 | 0 | 0 |

**Supplementary Table S5.** Distribution across UCCs of OTUs indicated as the most closely related to bacterial isolates (Supplementary Table S3). Only OTUs showing similarity of at least 97% are reported (Supplementary Data S3).

| **Strain ID** | **Closest species** | **OTU ID** | **Similarity (%)** | **LeAB (RA%)** | **NoAB (RA%)** | **ArAB (RA%)** |
| --- | --- | --- | --- | --- | --- | --- |
| **K1210** | *Pseudoroseomonas aestuarii* | Otu44 | 99.3 | 1.31  ± 0.30 | 0 | 3.23  ± 1.44 |
| **K7311** | *Pannonibacter phragmitetus* | Otu62 | 99.7 | 0.03  ± 0 | 0.45  ± 0.05 | 0 |
| **K7811** | *Agromyces indicus* | Otu9 | 99.7 | 0.61  ± 0.09 | 6.24  ±1.08 | 26.81  ±7.35 |
| **K1431** | *Agromyces indicus* | Otu9 | 99.7 | 0.61  ± 0.09 | 6.24  ±1.08 | 26.81  ±7.35 |
| **K1432** | *Agromyces arachidis* | Otu9 | 97.3 | 0.61  ± 0.09 | 6.24  ±1.08 | 26.81  ±7.35 |
| **K1240** | *Microbacterium hydrocarbonoxydans* | Otu11 | 97.0 | 0.06  ± 0.01 | 4.36  ± 0.43 | 1.48  ± 0.66 |

**Supplementary Table S6.** Parameters of the co-occurrence networks of the three UCCs, namely LeAB, NoAB and ArAB, respectively, computed considering all the bacterial OTUs (cyanobacteria host and heterotrophs bacteria).

**Supplementary Table S7.** Parameters of the co-occurrence networks of the three UCCs, namely LeAB, NoAB and ArAB, respectively, computed excluding OTUs of cyanobacteria host.
